# TPTE, a testis-specific PTEN family member, drives spermatogenesis via PI(4,5)P_2_ synthesis

**DOI:** 10.1038/s41419-026-08614-3

**Published:** 2026-03-25

**Authors:** Xu Chen, Tianye Wang, Haiqian Wu, Xiangzheng Zhang, Hao Chen, Longsheng Zhang, Yan Zhu, Xingyu Zhang, Bing Wang, Yueshuai Guo, Chenghao Situ, Ying Zheng, Yan Li, Xuejiang Guo, Hui Zhu

**Affiliations:** 1 Department of Histology and Embryology, School of Basic Medical Sciences, State Key Laboratory of Reproductive Medicine and Offspring Health, Nanjing Medical University, Nanjing, China; 2 School of Rehabilitation Science and Physical, Nanjing Normal University of Special Education, Nanjing, China; 3 Department of Histology and Embryology, School of Medicine, Yangzhou University, Yangzhou, China; 4 Department of Clinical Laboratory, Sir Run Run Hospital, Nanjing Medical University, Nanjing, China

**Keywords:** Phosphorylation, Spermatogenesis, Lipid signalling, Microtubules

## Abstract

Phosphatidylinositol(4,5) bisphosphate (PI(4,5)P_2_) is the most abundant phosphoinositides species and plays regulatory roles in spermatogenesis. However, the role of the phosphatases that regulate the production of PI(4,5)P_2_ during spermatogenesis has not been well studied. Here, we found that transmembrane phosphatase with tensin homology (TPTE), a testis-specific phosphatase and tensin homolog (PTEN) family member that catalyzes the generation of PI(4,5)P_2_, is important for spermatogenesis. The expression of *TPTE* is abnormally down-regulated in the testes of patients with non-obstructive azoospermia (NOA). In mouse, TPTE-deficiency reduced sperm count and decreased sperm motility, due to abnormal DNA double-strand break (DSB) repair in spermatocytes and flagellar defects in spermatids. TPTE catalyzes the generation of PI(4,5)P_2_, inhibiting the protein kinase B/mammalian target of rapamycin (AKT/mTOR) signaling pathway. During spermiogenesis, TPTE regulates AKT/mTOR-dependent translation of negative microtubule regulator PDZ and LIM domain 1 (PDLIM1) during the development of flagella. In meiosis, TPTE regulates the protein expression of RAD50 double strand break repair protein (RAD50), and promotes homologous recombination initiation. Collectively, our findings establish the important role of TPTE during spermatogenesis, providing new insights into the regulation of PI(4,5)P_2_ generation in male reproduction.

## Introduction

The development of germ cells into sperm is a complex process called spermatogenesis, involving mitosis of spermatogonia, meiosis of spermatocytes and spermiogenesis of spermatids [1, 2]. During meiosis, homologous chromosomes (HR) undergo recombination at DNA double-strand break (DSB) sites to generate new combinations of alleles on chromosomes. Single-stranded DNA binding proteins and recombinant enzymes are recruited to DSB sites to promote homologous recombination [3–5]. In spermiogenesis, the haploid round spermatids undergo dramatic morphological changes, such as nuclear condensation, flagellar assembly, and acrosomal formation, to develop into tadpole-shaped sperm [6, 7]. Defects in meiosis and/or spermiogenesis may cause abnormalities in sperm including reduced sperm count, decreased motility and sperm malformation, ultimately affecting male fertility [8].

Phosphoinositides (PIPs), which account for 1% of the total phospholipids, are a minor component of the lipid bilayer that makes up the plasma membrane [9]. Phosphoinositide kinases and phosphatases can produce localized pools of PIPs in different places in the cell [10]. PI(4,5)P_2_ is the most abundant among all seven PIPs species. As a critical second messenger, it plays regulatory roles in diverse cellular functions including cytoskeletal linkage, regulation of ion channels, and intracellular trafficking [9–11]. The cellular PI(4,5)P_2_ can be synthesized by dephosphorylation of phosphatidylinositol(3,4,5)-trisphosphate (PI(3,4,5)P_3_) [12]. However, the phosphatase that generates PI(4,5)P_2_ has not yet been well studied in spermatogenesis.

Phosphatase and tensin homolog (PTEN), which belongs to the PTEN family, is a well-known PI(3,4,5)P_3_ phosphatase to produce PI(4,5)P_2_, and is widely expressed in various tissues [13]. PTEN regulates activation of primordial follicles and its deficiency leads to premature ovarian failure in female mice [14, 15]. In male germ cells, PTEN regulates the maintenance of spermatogonial stem cells, but is dispensable for the subsequent processes of meiosis and spermiogenesis in mice [16], which might be regulated by the other members of the PTEN family, transmembrane phosphatase with tensin homology (TPTE) or transmembrane phosphoinositide 3-phosphatase and tensin homolog 2 (TPTE2). TPTE2 exists in human but not mouse [17–19]. TPTE is conserved between human and mouse and is specifically expressed in the testes [20]. TPTE, a germ cell-specific protein, contains phosphatase and C2 domains similar to PTEN, and additional transmembrane domains [21, 22]. Kawai et al. showed that deficiency of TPTE could affect the distribution of PI(4,5)P_2_ in the mouse sperm tail, and impair mouse sperm hyperactivation after capacitation [23]. However, the enzyme activity and function of TPTE in spermatogenesis are still not known.

In this study, we demonstrated that TPTE, as a germ cell-specific protein, can catalyze the production of PI(4,5)P_2_, and deficiency of TPTE led to male subfertility, with reduced sperm count and motility, and increased sperm tail malformations. TPTE suppressed the PI(4,5)P_2_-mediated protein kinase B/mammalian target of rapamycin (AKT/mTOR) signaling pathway to participate in spermatogenesis. TPTE regulates RAD50 double strand break repair protein (RAD50) expression to initiate homologous recombination during meiosis, and prevents overtranslation of PDZ and LIM domain 1 (PDLIM1) in spermatids and facilitates flagellar formation.

## Results

### TPTE is a germ cell-specific functional phosphatase and catalyzes PI(3,4,5)P_3_ to produce PI(4,5)P_2_

**Fig. 1:**
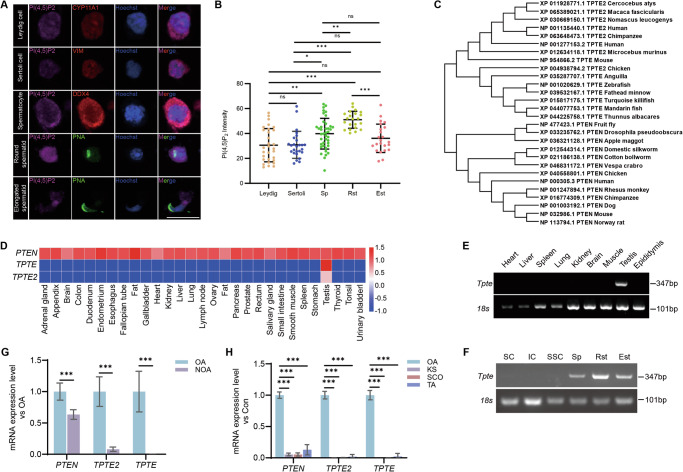
Expression of PI(4,5)P_2_ and TPTE in testis. **A** Immunofluorescence co-staining of PI(4,5)P_2_ (magenta), and CYP11A1 (Leydig cell), VIM (Sertoli cell), DDX4 (germ cell) or PNA (acrosome) in testicular cells. DDX4 (red) and PNA (green) was used to distinguish PNA negative spermatocyte and PNA positive spermatid. Nuclei were stained with Hoechst (blue). Bar, 10 μm. **B** The average intensity of PI(4,5)P_2_ in germ cells and various types of somatic cells (*n* = 30 for Leydig, *n* = 26 for Sertoli, *n* = 40 for spermatocyte, *n* = 22 for round spermatid, *n* = 23 for elongated spermatid; one-way ANOVA with Dunnet´s test). Sp, Spermatocytes; Rst, Round spermatids; Est, Elongated spermatids. **C** Phylogenetic tree showing the evolutionary distance between PTEN family members among different animals. **D** mRNA expression levels of PTEN family members in multiple human tissues. Expression level of *Tpte* mRNA in multiple mouse tissues (**E**) and different types of testicular cells (**F**) by RT-PCR. 18S was used as a loading control. SC, Sertoli cells; IC, Interstitial cells; SSC, Spermatogonial stem cells; Sp, Spermatocytes; Rst, Round spermatids; Est, Elongated spermatids. **G**, **H** Expression of PTEN family members in testis of various infertile patients (G: *n* = 3 for OA, *n* = 3 for NOA; H: *n* = 5 for OA, *n* = 5 for KS, *n* = 5 for SCO, *n* = 5 for TA; one-way ANOVA with Dunnet´s test). OA, obstructive azoospermia; NOA, non-obstructive azoospermia; KS, Klinefelter syndrome; SCO, Sertoli cell-only syndrome; TA, testis atrophy. ns, not significant; *P < 0.05, **P < 0.01, ***P < 0.001.

PI(4,5)P_2_ is the most abundant form of PIPs, and its distribution in testis is not known. In order to clarify the distribution of PI(4,5)P_2_ in spermatogenic cells, we performed immunofluorescence analysis of PI(4,5)P_2_, and co-stained Cytochrome P450 family 11 subfamily A member 1 (CYP11A1), Vimentin (VIM), DEAD box helicase 4 (DDX4) and Peanut Agglutinin (PNA) as markers for Leydig cells, Sertoli cells, germ cells and spermatids, respectively. Spermatocytes are the largest DDX4 positive germ cells with large nuclei, and can be easily distinguished from spermatogonia in size. The result showed that PI(4,5)P_2_ was localized in the cytoplasm and nucleus of various testicular cells, with enrichment in germ cells (spermatocytes and spermatids) relative to Leydig cells and Sertoli cells, and the highest level in round spermatids (Fig. 1A, B), suggesting that PI(4,5)P_2_ may play important roles in spermatogenesis. PTEN catalyzes the production of PI(4,5)P_2_ [22, 24]. The PTEN family consists of PTEN, TPTE and TPTE2. Evolutionary analysis revealed that PTEN was highly conserved in many species, from silkworm to human. TPTE exists in human, mouse and fish, while TPTE2 exists only in primates (Fig. 1C). In human [25], *PTEN* is widely expressed in many tissues, while *TPTE* and *TPTE2* are specifically expressed in testis (Fig. 1D). Interestingly, *TPTE* had highest expression in testis compared with *PTEN* and *TPTE2* (Fig. 1D). We analyzed the expression of *Tpte* by reverse transcription polymerase chain reaction (RT-PCR), and found that *Tpte* was also a testis-specific gene (Fig. 1E). In mouse testicular cells, *Tpte* is expressed in germ cells including spermatocytes, round and elongated spermatids but not somatic cells (Fig. 1F). *Tpte* may regulate the developmental processes of spermatocytes and spermatids.

As a conserved testis-specific gene, *Tpte* may play testis-specific functions and be involved in male infertility. Non-obstructive azoospermia (NOA) is the most severe type of male infertility. We analyzed the testis RNA-seq data of NOA published by Tang et al.[26], and found that the expression of *PTEN*, *TPTE*, and *TPTE2* were all reduced in patients with NOA, with *TPTE* decreased most significantly (Fig. 1G). To further evaluate the roles of TPTE in male infertility, we analyzed the RNA-seq data of Klinefelter syndrome (KS), Sertoli cell-only syndrome (SCO) and testicular atrophy (TA) published by Willems et al[27]. (Fig. 1H), and found that *TPTE* was abnormally downregulated or absent in all those testis tissues. Willems et al.’s pathological examination showed existence of germ cells in 9-47% of the seminiferous tubules in the TA samples (sample 1–4) [27]. The absence and decreased *TPTE* expression in the testicular tissues is ascribed not only to the reduced number of germ cells in the testes, but also the diminished expression in germ cells. These results suggested that *TPTE* may play important functions in spermatogenesis and male fertility.

**Fig. 2:**
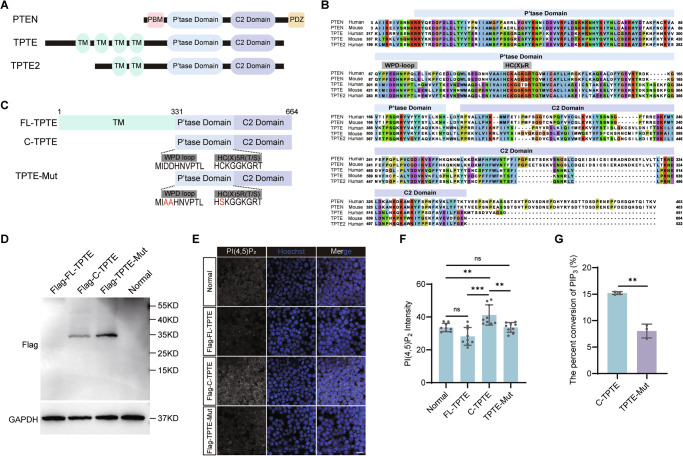
TPTE dephosphorylated PI(3,4,5)P_3_ to PI(4,5)P_2_. **A** Schematic representation of the domain structures of PTEN, TPTE and TPTE2. TM, transmembrane segments; PBM, photobiomodulation; PDZ, PSD-95/Dlg/ZO-1 (PDZ) binding motif. **B** Amino acid sequence alignment of phosphatase domains and C2 domains of human and mouse PTEN family members. **C** Schematic representation of FL-TPTE, C-TPTE and TPTE-Mut. FL-TPTE: full-length of TPTE; C-TPTE: C-terminal region of 331-664aa; TPTE-Mut: Mutate aspartate (D) to alanine (A) in WPD-loop domain and cysteine (C) to serine (S) in the HC(X)_5_R motif. **D** Flag-FL-TPTE, Flag-C-TPTE and Flag-TPTE-Mut overexpressed 293 T cells were detected by Western blot. (**E**) Immunofluorescence staining of PI(4,5)P_2_ in Flag-FL-TPTE, Flag-C-TPTE and Flag-TPTE-Mut overexpressed 293 T cells. White, PI(4,5)P_2_; blue, Hoechst. Bar, 50 μm. **F** The average intensity of PI(4,5)P_2_ in overexpressed 293 T cells (*n* = 9 for cell cross sections per group; one-way ANOVA with Dunnet´s test). **G** Flag-C-TPTE and Flag-TPTE-Mut reacted with PI(3,4,5)P_3_, and the conversion ratio of PI(3,4,5)P_3_ was calculated based on released phosphate by malachite green (*n* = 3, two-tailed Student’s *t*-test). ns, not significant; **P < 0.01, ***P < 0.001.

Protein tyrosine phosphatases (PTPs) are defined by the active site signature motif HC(X)_5_R called the P-loop [28]. Catalysis by PTPs is also regulated by a conserved loop, consisting of about a dozen residues called the WPD (Trp-Pro-Asp) loop [29, 30]. TPTE, TPTE2 and PTEN all have a phosphatase domain (Fig. 2A), but their phosphatase domains have different sequences of WPD loop and HC(X)_5_R, which might affect the catalytic functions (Fig. 2B). With the PTP domain, TPTE is expected to catalyze the conversion of PI(3,4,5)P_3_ to PI(4,5)P_2_ without the need of cofactors. HEK293T, a widely used cell line for biochemical studies [31–33], was used to evaluate the catalytic function of mouse TPTE to generate PI(4,5)P_2_. We overexpressed full-length TPTE, truncated TPTE (331-664aa) with C-terminal region containing the phosphatase and C2 domains, and mutated TPTE (331-664aa) (TPTE-Mut) with the aspartate of WPD-loop converted to alanine, and the cysteine in the HC(X)_5_R motif to serine [34] (Fig. 2C). The results showed that full-length TPTE was not successfully overexpressed in HEK293T cells (Fig. 2D). With successful TPTE (331-664aa) overexpression (Fig. 2D), we found increased production of PI(4,5)P_2_ as measured by immunofluorescence (Fig. 2E-F). Meanwhile, TPTE was immunoprecipitated for in vitro dephosphorylation assay to convert PI(3,4,5)P_3_ to produce PI(4,5)P_2_. The conversion of PI(3,4,5)P_3_ to PI(4,5)P_2_ releases a phosphate acid, which also indicates the catalytic activity of the phosphatase. Malachite green test was used to measure the level of phosphate acid (Fig. 2G). These results showed that compared with the TPTE (331-664aa) group, less phosphate acid could be measured in the TPTE-Mut group, supporting the important functions of WPD-loop and HC(X)_5_R motif in catalytic function of phosphatase domain in TPTE.

### Loss of TPTE impairs sperm quantity and quality and leads to subfertility in male mice

**Fig. 3:**
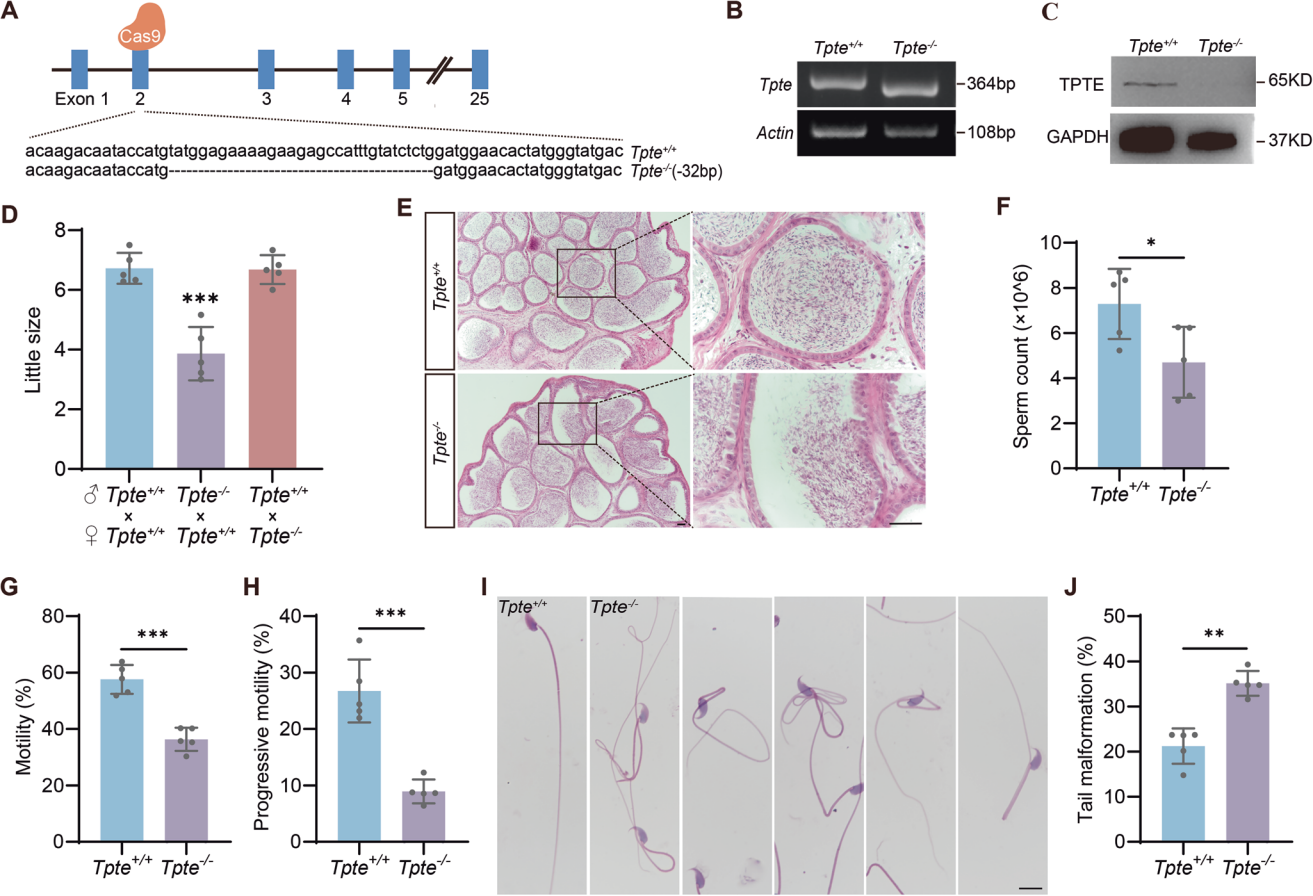
Loss of TPTE damages male fertility. **A** Schematic diagram of targeting strategy by CRISPR/Cas9. Sanger sequencing results show a 32-bp deletion in exon 2. **B** Genotyping confirmed deletion of exons 2 in *Tpte*^*−/*^^−^ mice. **C** Western blot analysis of testicular TPTE expression. GAPDH was used as an internal control. **D** *Tpte*^*−/−*^ and *Tpte*^*+/+*^ mice were tested for fertility (*n* = 5, one-way ANOVA with Dunnet´s test). (**E**) Histological structure of the cauda epididymis by H&E. Bars, 50 μm. **F** Analysis of sperm count (*n* = 5, two-tailed Student’s *t* test). Computer-assisted sperm analysis (CASA) of sperm motility (**G**) and progressive motility (**H**) (*n* = 5, two-tailed Student’s *t* test). H&E stained sperm morphology (**I**) and the statistics of sperm tail malformation (**J**) (*n* = 5, two-tailed Student’s *t* test). Bar, 10 μm. *P < 0.05, **P < 0.01, ***P < 0.001.

To study the in vivo function of germ cell-specific TPTE, we generated *Tpte* knockout mice using clustered regularly interspaced short palindromic repeats (CRISPR)-CRISPR-associated protein 9 (Cas9) technology with two single-guide RNAs. Genotyping PCR and DNA sequencing of the mice showed that the knockout mice exhibited a 32-bp deletion (Fig. 3A, B), resulting in frameshift mutations (Appendix Figure S1A, B). Quantitative real-time PCR (qRT-PCR) analysis revealed significantly lower mRNA expression in the testis of knockout mice (Appendix Figure S1C), presumably degraded via the nonsense mediated decay pathway. Western blot analysis showed absence of TPTE protein expression in the testis of the mutated mice (Fig. 3C), indicating successful generation of TPTE-deficient mouse model (*Tpte*^*−/−*^ mice). We tested the male fertility of adult TPTE-deficient mice by mating them with fertile adult wild-type mice (*Tpte*^*+/+*^ mice) for up to 5 months. The results showed that the average litter size of *Tpte*^*−/−*^ male mice was significantly decreased (Fig. 3D), suggesting that TPTE is important for male fertility.

In order to explore the reasons for the decreased fertility of *Tpte*^*−/−*^ male mice, we analyzed the cauda epididymis of adult male mice. There was no abnormality in the epididymis size and epididymis/body weight between *Tpte*^*+/+*^and *Tpte*^*−/−*^ mice (Appendix Figure S2). Hematoxylin and eosin (H&E) staining of the cauda epididymis showed a lower sperm density (Fig. 3E). We further evaluated sperm parameters in the caudal epididymis. The result showed a significantly reduced sperm count in *Tpte*^*−/−*^ mice compared to the control mice (Fig. 3F). Functional analysis of *Tpte*^*−/−*^ sperm revealed lower motility and progressive motility (Fig. 3G, H). The morphological analysis showed that the *Tpte*^*−/−*^ sperm exhibited apparent normal head shape, but abnormally curled and folded tail (Fig. 3I). Statistical results consistently indicated a higher rate of sperm tail deformity in *Tpte*^*−/−*^ mice (Fig. 3J). Therefore, TPTE plays important roles in spermatogenesis, affecting sperm quantity and quality.

### TPTE suppresses the expression of RAD50 to affect DSB repair through AKT/mTOR signaling pathway

**Fig. 4:**
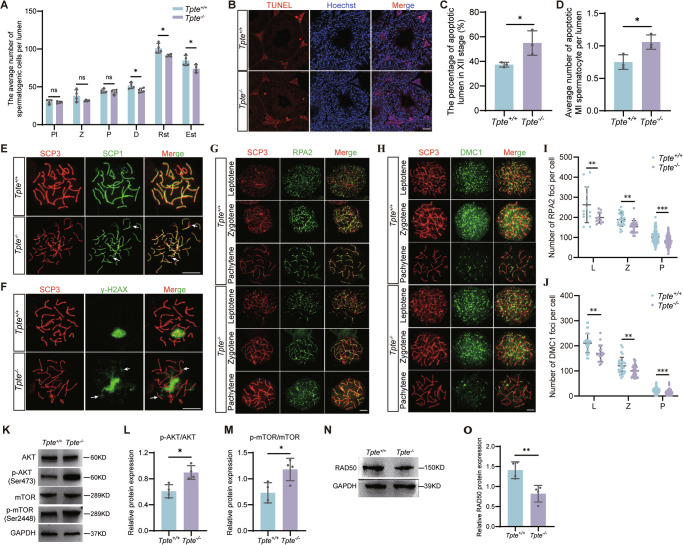
TPTE is involved in DSB repair in meiosis. **A** The distribution of the number of spermatogenic cells per tubule (*n* = 4, two-tailed Student’s *t*-test). The number of germ cells in the lumen of Ⅶ-Ⅷ stage and XI stage was calculated in 5 sections of different parts of testis. Pl, Preleptotene; Z, Zygotene; P, Pachytene; D, Diplotene; Rst, Round spermatid; Est, Elongated spermatid. **B** TUNEL staining of testis. Red, apoptotic cell; blue, Hoechst. Bar, 20 μm. The percentage of apoptosis lumens (**C**) and average number of apoptotic MI spermatocytes (**D**) in Ⅻ stage of testis. (*n* = 3, two-tailed Student’s *t* test). (**E**) Double immunostaining for SCP1 (green) and SCP3 (red) identifies synaptonemal complexes in pachytene spermatocytes (*n* = 3). White arrow, defective synaptonemal complexes. Bar, 10 μm. (**F**) Chromosome spreads of pachytene spermatocytes were stained for γ-H2AX (green) and SCP3 (red) (*n* = 3). White arrow, residual γ-H2AX signals at autosomal locations. Bar, 10 μm. **G**, **J** Co-immunostaining of SCP3 (red), and RPA2 (**G**) or DMC1 (**H**) (green) in spermatocytes (n = 3, two-tailed Student’s *t* test). The average number of foci of RPA2 (**I**) and DMC1 (**J**) in each spermatocyte. Bar, 5 μm. (**K**) Western blot analysis of AKT, p-AKT (Ser473), mTOR, p-mTOR (Ser2448) in *Tpte*^*+/+*^ and *Tpte*^*−/−*^ testes. The quantification of phosphorylation levels of AKT (**L**) and mTOR (**M**) (*n* = 4, two-tailed Student’s *t* test). Western blot (**N**) and quantification results (**O**) of RAD50 in *Tpte*^*+/+*^ and *Tpte*^*−/−*^ testes (*n* = 4, two-tailed Student’s *t* test). ns, not significant, *P < 0.05, **P < 0.01, ***P < 0.001.

TPTE deficiency reduced sperm counts. To identify the exact stage at which the number of germ cells starts to decrease, we further analyzed the phenotype of testis. We found no change in testis size or testis/body weight ratio (Appendix Figure S3A, B). Periodic acid-Schiff (PAS) staining showed similar thickness (Appendix Figure S3C) and combinations of different stages of male germ cells (Appendix Figure S3D) in *Tpte*^*−/−*^ testis compared with those of the control group. Quantitation of different stages of germ cells showed that the counts started to decrease from diplotene spermatocytes (Fig. 4A). Abnormalities in meiosis might lead to apoptosis. We performed terminal deoxynucleotidyl transferase dUTP Nick-End Labeling (TUNEL) staining of the *Tpte*^*−/−*^ testis. The counting analysis revealed significantly increased percentage of lumens with apoptotic metaphase I spermatocytes in XII stage, and increased average number of apoptotic metaphase I spermatocytes per lumen in XII stage (Fig. 4B–D).

Cell counting showed important roles of TPTE for meiotic progression. During meiosis, homologous recombination is a complex process initiated by DSB formation followed by synapsis of homologous chromosomes and DSB repair, and crossing over [35]. We evaluated synapsis of homologous chromosomes, using synaptonemal complex protein 1 (SCP1) and synaptonemal complex protein 3 (SCP3), the marker proteins of the lateral and central elements of synaptonemal complex. Chromosome spread experiments showed that SCP1 was found to be absent and not fully co-located with SCP3 in *Tpte*^*−/−*^ pachytene spermatocyte, suggesting abnormal formation of synaptonemal complex (Fig. 4E). Meiotic DSBs are marked by γ-H2AX at leptotene stage, and are repaired on autosomes during pachytene stage [36]. We stained γ-H2AX, and found that in *Tpte*^*+/+*^ pachytene spermatocytes, γ-H2AX signals were restricted to the sex chromosomes, whereas in *Tpte*^*−/−*^ pachytene spermatocytes, residual γ-H2AX signals were still observed on the autosomes (Fig. 4F), suggesting that DNA repair may be abnormal. During homologous recombination, the complex of Replication Protein A 2 (RPA2) binds to and protects the single-stranded DNA overhangs from nucleolytic degradation, and recruits DNA meiotic recombinase 1 (DMC1) to catalyze homology search [37, 38]. We detected RPA2 and DMC1, and the results showed that in the control spermatocytes, the RPA2 (Fig. 4G) and DMC1 (Fig. 4H) foci localize at DSB sites on chromosome axes from leptotene stage to pachytene stage. The average numbers of RPA2 (Fig. 4G, I) and DMC1 (Fig. 4H, J) foci in *Tpte*^*−/−*^ spermatocytes were significantly reduced, indicating disrupted homologous recombination. The absence of TPTE affects DSB repair and formation of synaptonemal complex in meiosis, which leads to increased apoptosis of some spermatocytes and decreased the number of round and elongated spermatids. This cascade of defects constitutes a reason underlying the reduction in sperm count in TPTE-deficient male mice.

PI(4,5)P_2_, the product of TPTE, can phosphorylate and activate the downstream AKT/mTOR signaling pathway. We detected phosphorylation of AKT and mTOR in testis. The results showed significantly increased phosphorylation level of AKT (S473) and mTOR (S2448) in *Tpte*^*−/−*^ testes without the change at their protein expression level (Fig. 4K–M). Piscitello et al. found that AKT overaction inhibits the expression of RAD50 to suppress DNA repair [39]. RAD50 is a key protein reported to initiate DSB repair [40, 41]. We examined protein expression using Western blot, and validated that the expression of RAD50 was reduced in *Tpte*^*−/−*^ testis (Fig. 4N, O). Overall, the TPTE deficiency leads to down-regulate the expression of RAD50, abnormal DSB repair and increased spermatocyte apoptosis, resulting in a decrease in spermatid counts.

### TPTE affects the flagellar formation during spermatid differentiation

**Fig. 5:**
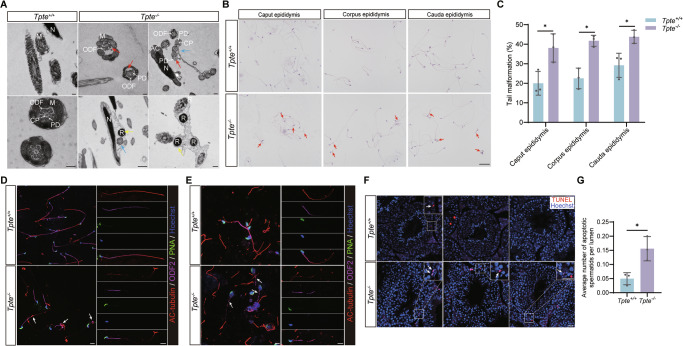
Morphological analysis of sperm tail abnormalities in epididymis. **A** Ultrastructure of *Tpte*^*+/+*^ and *Tpte*^*−/−*^ sperm by transmission electron microscope. Blue arrow, multiple cross sections of the tail wrapped in the same cell; red arrow, misaligned or absent “9 + 2” microtubules and peripheral dense fiber; yellow arrow, high-density particles in sperm. M, mitochondrial sheath; N, nucleus; AC, acrosome; ODF, outer dense fibers; CP, central microtubules; PD, peripheral microtubule doublets; R, residual body. Bar, 500 nm. H&E staining (**B**) and the statistics of tail malformation (**C**) of spermatozoa in caput, corpus and cauda epididymis (*n* = 3, two-tailed Student’s *t* test). Red arrow, tail malformation. Bar, 50μm. Immunofluorescence staining of AC-Tubulin (red), ODF2 (magenta), PNA (green) and Hoechst (blue) in sperm from cauda epididymis (**D**) and elongated spermatids from testis (**E**) (n = 3). White arrow, abnormal sperm and spermatids. Bar, 20 μm. (**F**) TUNEL staining of testis. Red, apoptotic cell; blue, Hoechst; white arrow, apoptotic spermatids. Bar, 20 μm. **G** The average number of apoptotic spermatids in testis. (*n* = 3, two-tailed Student’s *t* test). *P < 0.05.

TPTE deficiency led to increased abnormalities of sperm tails and reduced motility and progressive motility. In order to find the reason for sperm motility decline, we performed ultrastructure analysis of sperm from cauda epididymis, and found that the *Tpte*^*+/+*^ sperm tail had normal and orderly structure, but the *Tpte*^*−/−*^ sperm tails were abnormal, with multiple tail cross-sections wrapped in one cell membrane, disordered or absent arrangement of “9 + 2” microtubules and/or peripheral dense fibers (Fig. 5A). We also found that many high-density particles were encapsulated in cell membrane without a nucleus (Fig. 5A). Sperm are produced in the testis and transported to the epididymis for maturation and storage. We analyzed the morphology of sperm from caput, corpus, and cauda epididymis, respectively. Sperm in the caput epididymis and corpus epididymis showed the same tail deformity as sperm in the cauda epididymis (Fig. 5B, C), suggesting that sperm malformations in *Tpte*^*−/−*^ male mice may be caused by abnormal sperm formation in the testis. Immunofluorescence staining of acetylated alpha-tubulin (AC-Tubulin) and outer dense fiber of sperm tails 2 (ODF2) was performed on mature sperm and elongated spermatids, and the results showed reduced level and discontinuous distribution of AC-Tubulin and ODF2 in *Tpte*^*−/−*^ sperm and elongated spermatids (Fig. 5D, E). We further performed TUNEL staining, and observed an increased number of apoptotic elongated spermatids in the *Tpte*^*−/−*^ testis (Fig. 5F, G), suggesting that some abnormally differentiated spermatids undergo apoptosis and affect the number of sperm.

TPTE plays an important role in the flagella development of spermatids. The absence of TPTE protein leads to decreased sperm count and structural defects of sperm flagella, which is the cause of decreased sperm motility.

### TPTE regulates PDLIM1 translation by PI(4,5)P_2_-mediated AKT/mTOR pathway in flagellar formation

**Fig. 6:**
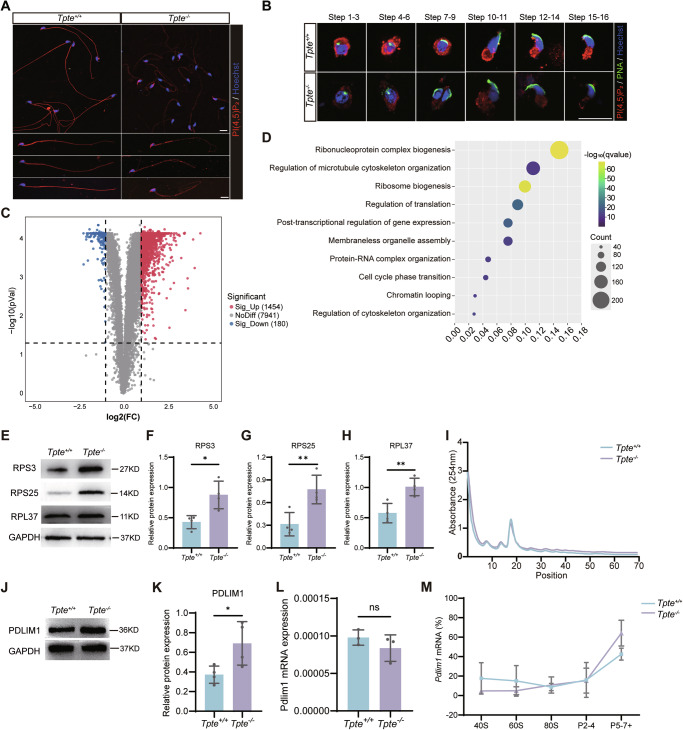
TPTE dephosphorylates PI(3,4,5)P_3_ to regulate translation in spermiogenesis. Immunofluorescence analysis of PI(4,5)P_2_ (red), PNA (green) and Hoechst (blue) in sperm from caudal epididymis (**A**) and spermatids from testis (**B**) (*n* = 3). Bar, 20 μm. **C** Volcano plot of differentially expressed proteins in round spermatids. **D** GO terms enriched in upregulated proteins in *Tpte*^*−/−*^ round spermatids. Western blot (**E**), and quantification of RPS3 (**F**), RPS25 (**G**) and RPL37 (**H**) in *Tpte*^*+/+*^ and *Tpte*^*−/−*^ round spermatids (*n* = 4, two-tailed Student’s *t* test). (**I**) The absorbance of ribosome profiles in *Tpte*^*+/+*^ and *Tpte*^*−/−*^ round spermatids. Western blot (**J**) and quantification (**K**) of PDLIM1 expression in *Tpte*^*+/+*^ and *Tpte*^*−/−*^ round spermatids (*n* = 4, two-tailed Student’s *t* test). (**L**) The level of total *Pdlim1* mRNA in round spermatids (*n* = 3, two-tailed Student’s *t* test). **M** Polysome distribution of *Pdlim1* mRNAs in different fractions of ribosome profiling of round spermatids. ns, not significant, *P < 0.05, **P < 0.01.

TPTE catalyzes the conversion of PI(3,4,5)P_3_ to PI(4,5)P2. As PI(3,4,5)P_3_ cannot be directly detected by commercially available antibody, we evaluate the changes of PI(4,5)P_2_ levels. The results showed a lower level of PI(4,5)P_2_ with discontinuous distribution in the *Tpte*^*−/−*^ sperm flagellum (Fig. 6A). In testis, PI(4,5)P_2_ was significantly reduced in *Tpte*^*−/−*^ spermatids (Fig. 6B), indicating defect in dephosphorylation of PI(3,4,5)P_3_ to PI(4,5)P_2_ in the absence of TPTE.

The TPTE deficiency caused sperm flagellar abnormalities. To identify proteins affecting flagellar formation, we isolated round spermatids from testis and performed quantitative proteomics analysis. This analysis identified 1454 upregulated and 180 down-regulated proteins in *Tpte*^*−/−*^ round spermatids (Fig. 6C, Appendix Table S2) with majority of the differentially regulated proteins upregulated. Gene Ontology analysis of the upregulated proteins showed enrichment in ribosome biogenesis, regulation of microtubule cytoskeleton organization and regulation of translation (Fig. 6D, Appendix Table S3). AKT/mTOR signaling regulate the activity of ribosome and protein translation. We observed increased expression of ribosomal protein S3 (RPS3), ribosomal protein S25 (RPS25) and ribosomal protein L37 (RPL37) in the quantitative proteome data of *Tpte*^*−/−*^ round spermatids (Fig. 6E–H), which might further enhance protein translation. To evaluate the ribosome activity, we performed ribosome peak profile analysis, and observed increase in the polysome peak, suggesting active polysome translation in *Tpte*^*−/−*^ round spermatids (Fig. 6I). In the upregulated proteins in *Tpte*^*−/−*^ round spermatids, we focused on negative regulators of cytoskeleton, and found that the protein level of the microtubule negative regulators PDLIM1 in *Tpte*^*−/−*^ round spermatids was increased (Fig. 6J-K). Nevertheless, we found that the total mRNA level of *Pdlim1* exhibited no change in *Tpte*^*−/−*^ round spermatids (Fig. 6L), indicating activated translation. Further polysome profiling analysis revealed the *Pdlim1* mRNA in the heavy polysome was upregulated, indicating increased translation efficiency of PDLIM1 in *Tpte*^*−/−*^ round spermatids (Fig. 6M). Collectively, these results suggested that TPTE affected protein translation through PI(4,5)P_2_-mediated AKT/mTOR signaling pathway to regulate flagellum development during spermiogenesis.

## Discussion

We found that TPTE, a germ cell-specific phosphatase, can dephosphorylate PI(3,4,5)P_3_ to produce PI(4,5)P_2_. TPTE deficiency resulted in male subfertility, characterized by decreased sperm count, decreased motility and increased tail deformity. In meiosis, TPTE regulates RAD50 expression via AKT/mTOR pathway to affect DSB repair. During spermiogenesis, TPTE participates in PI(4,5)P_2_/AKT/mTOR-mediated PDLIM1 translation and regulates flagellar development in spermatids.

In meiosis, we found that TPTE deficiency activated phosphorylation of AKT Ser473, affected DSB repair and increased apoptosis of spermatocytes. In spermatocytes MRE11-RAD50-NBS1 (MRN) complex recognizes the DNA break site and initiates homologous recombination to promote DSB repair [40–43]. Excessive activation of AKT Ser473 phosphorylation was reported to inhibit the expression of the MRN complex in colon cancer cells [39, 43, 44]. We found that TPTE deficiency led to decrease of RAD50 during meiosis, which might cause reduced DSB formation, impaired DSB end resection and meiotic defects.

Our analysis of PI(4,5)P_2_ showed that it exhibits higher level in round spermatids, and TPTE can catalyze the dephosphorylation of PI(3,4,5)P_3_ to produce PI(4,5)P_2_. In *Tpte*^*−/−*^ mice, the sperm flagella showed different degrees of curling and folding, and electron microscope results showed that sperm had multiple tail cross-sections wrapped in the one cell membrane caused deformity of peripheral dense fibers and microtubules. Kawai et al. generated TPTE-deficient mice model through knock-in technology and demonstrated that PI(4,5)P_2_ exhibited abnormal distribution pattern affecting sperm capacitation in TPTE-deficient mice [23]. A growing body of evidence suggest that membrane PI(4,5)P_2_ can regulate actin-binding or microtubule organization proteins to control the cytoskeleton network [11], and it can regulate F-actin polymerization to affect sperm hyper-activated motility in sperm capacitation [45]. Additionally, PI(4,5)P_2_ deficiency can lead to abnormality in sperm motility-related structures and male fertility in Drosophila [46, 47]. In mice lacking TPTE, we found that PI(4,5)P_2_ was significantly reduced in spermatids, and appeared at a reduced level with uneven distribution in mature sperm flagella. TPTE can participate in sperm flagellum development by directly regulating the content of PI(4,5)P_2_.

Spermatid differentiation requires the orderly expression of proteins. Our experimental results found that the absence of TPTE prevented the dephosphorylation of PI(3,4,5)P_3_, leading to hyperphosphorylation of downstream AKT/mTOR in round spermatids, thereby activating protein translation. The homeostasis of ribosome is important for successful spermiogenesis. Our previous studies reported the translation by spermatid-specific ribosome (Ribosome^ST^) is essential for sperm protein translation and male fertility [48]. While block of autophagic degradation of intracellular ribosomes in ARMC3-deficient spermatids can result in spermiogenesis defects [49]. We found that *Tpte*^*−/−*^ round spermatids exhibited upregulation of ribosomal proteins and most of the differentially regulated proteins were upregulated. And *Pdlim1*, a negative regulator of microtubules, exhibited higher level of translation and expression and may contribute to the defects of sperm flagella assembly and male fertility.

This study has some limitations. As an important gene in spermatogenesis, it is not known whether there are any genetic mutations of *TPTE* in infertile men. It can be evaluated by analysis of peripheral blood from infertile patients by whole exome sequencing to identify potential disease-causing mutations of *TPTE*. For the functions of TPTE, further studies are needed to elucidate the mechanism by which TPTE regulates the expression of RAD50 in spermatocytes.

In summary, we found that germ-cell specific *TPTE* is important for male fertility and abnormally down-regulated in testis of infertile patients. TPTE deficiency can lead to decreased sperm count and motility, and increased tail malformation. TPTE catalyzes the generation of PI(4,5)P_2_ to inhibit the AKT/mTOR signaling pathway. In meiosis, TPTE regulates the expression of RAD50 and DSB repair. During spermiogenesis, TPTE maintains ribosome homeostasis to regulate flagellar development. Elucidation of its function and molecular mechanism in spermatogenesis has the potential to facilitate the development of targeted treatment strategies for male infertility.

## Materials and methods

### Animals

All animal experiments were approved by the Animal Experimentation Ethics Committee of Nanjing Medical University. *Tpte* knockout mice were generated on the C57BL6 background via Cas9/RNA-mediated gene targeting and were bred at the animal center of Nanjing Medical University (Nanjing, China). All the mice were housed under specific pathogen free condition with unlimited access to food and water. The constant room was maintained at the temperature of 22–24 °C with a light:dark cycle of 12:12. *Tpte* knockout mice (*Tpte*^*−/−*^) were designed as the experimental group and littermate wild type (*Tpte*^*+/+*^) mice as the control group. The *Tpte* gene was identified via PCR using the forward primer 5′-AGAACCTATACCAGACCTAGGGC-3′ and the reverse primer 5′-ATGTCCCAGGCTAGGTGGTAC-3′. The primers for *Actin* are forward primer 5′- AGATCAAGATCATTGCTCCTCCT-3′ and reverse primer 5′- ACGCAGCTCAGTAACAGTCC-3′. The PCR products were subjected to Sanger sequencing.

### RNA sequencing analysis

The RNA-seq data were obtained from the published studies by Tang et al. (GSE190752) [26] and Willems et al. (GSE200680) [27]. Tang et al.’s data included testicular samples from patients with secondary idiopathic NOA [26]. Willems et al.’s data included samples from those with adult SCO, TA (samples with a normal karyotype showing testicular fibrosis) and KS [27]. Samples from patients with OA were included as controls in both studies. And all testicular biopsy samples were evaluated by histological examination, and sequenced by Illumina platforms [26, 27]. The nf-core/RNAseq (v3.14.0) pipeline [50] was used for data processing. Briefly, raw reads were first assessed for quality using FastQC (v0.12.1) and then aligned to the human reference genome (GRCh37) with STAR (v2.6.1 d). Transcript-level expression was quantified using Salmon (v1.10.1). Gene-level counts were summarized and imported into DESeq2 (v1.44.0) in R (v4.4.1) for normalization and differential expression analysis.

Human RNA expression data of multiple tissues were derived from the Wang et al.‘s report [25]. Fragments per kilo base per million mapped reads (FPKM) were obtained and heatmaps were used to illustrate the relative expression levels of genes across tissues.

### Phylogenetic analysis

Orthologs of PTEN family were retrieved from the National Center for Biotechnology Information (NCBI) Orthologs. The sequence alignment was performed using the MEGA11 software with the MUSCLE method, and the *p* distance < 0.7. The phylogenetic tree of PTEN family proteins from various species was constructed using the neighbor-joining method with a bootstrap of 1000 by MEGA11 software [51, 52].

### RNA isolation, reverse transcription polymerase chain reaction (RT-PCR) and quantitative real-time PCR (qRT-PCR)

Total RNA was extracted from samples by using Trizol reagent (Invitrogen, 15596-026) according to the manufacturer’s instructions. mRNA was reverse-transcribed to cDNA using HiScript II Q Select RT SuperMix for qRT-PCR (Vazyme, R223). RT-PCR of cDNA was carried out using 2 × Phanta Max Master Mix (Vazyme, P515-01). The primers used in RT-PCR: *Tpte*-F1: 5′-AACTGGAGTGGACTCAGCTCC-3′; *Tpte*-R1: 5′-TGGATATGACAAACTGCTCCCG-3′; *Tpte*-R2: 5′-TCCATCCAGAGATACAAATGGCTCT-3′; *Tpte*-R3: 5′-CCATAGTGTTCCATCCATGGTATTGT-3′. qRT-PCR of cDNA was carried out using Taq Pro Universal SYBR qPCR Master Mix (Vazyme, Q712) with ABI Q5 real-time PCR System (Applied Biosystems, Thermo Fisher Scientific, MA, USA). The primers used in qRT-PCR: *Tpte* (forward primer 5′-TCGAGGGGTAGCTAAGCTGT-3′; reverse primer 5′-TTTGTGTCGTAGCCAGTGCT-3′), *18S* (forward primer 5′-CGCCGCTAGAGGTGAAATTCT-3′; reverse primer 5′-CGAACCTCCGACTTTCGTTCT-3′), *Pdlim1* (forward primer 5′- ATAGGAAGTGCCCACAACCG-3′; reverse primer 5′- TTGTTGGGATCCCCTTTCCC-3′). The gene expression levels were calculated by 2^(-ΔCT)^.

### Fertility tests

8–12-week-old *Tpte*^*−/−*^ and *Tpte*^*+/+*^ mice were mated with fertile wild-type mice for at least 5 months at a 2:1(female: male) ratio. Plugs were checked every morning, and the number of offspring born after pregnancy was recorded. Analysis of the average number of offspring was considered as a measure of fertility.

### Computer-Assisted Sperm Analysis

Sperm were squeezed out from the cauda epididymis and incubated in TYH (Toyoda, Yokoyama, Hoshi) Medium (Aibei, M2050) at 37°C for 5 min. Sperm count, motility and progressive motility of semen samples were assessed by computer-assisted semen analysis (CASA) using the IVOS II Sperm Analyzer (Hamilton Thorne, MA, USA).

### Histological analysis

Testes and epididymis from adult mice were fixed in modified Davidson’s fluid (30% formaldehyde, 15% ethanol, 5% glacial acetic acid, and 50% distilled H_2_O). The tissues were dehydrated with a gradient alcohol and embedded in paraffin and cut into 5 µm sections. Periodic acid-Schiff (PAS) (Biochannel, BP-DL031) and hematoxylin-eosin (H&E) (Biochannel, BP-DL001) staining were performed on sections according to the manufacturer’s protocol. Sperm from the caput epididymis, corpus epididymis, and cauda epididymis were transferred to slides and air-dried naturally. After fixation with 4% paraformaldehyde, sperm morphology was observed by H&E staining. The morphological structure of the tissue was observed under a microscope (Nikon, Tokyo, Japan).

### Quantification of spermatogenic cells

To quantify spermatogenic cells, five sections were randomly selected from each testicular tissue sample and stained with PAS, and the stages of seminiferous tubules were determined according to the literature [53]. Seminiferous tubules with a round lumen morphology at stages Ⅶ–Ⅷ and XI were selected for analysis. The pachytene spermatocytes, preleptotene spermatocytes, and round spermatids in stages VII–VIII were counted. The zygotene spermatocytes, diplotene spermatocytes, and elongated spermatids in stage XI were counted. Based on the statistical data, the average number of spermatogenic cells per seminiferous tubule was calculated.

### Electron microscopy

Sperm samples isolated from cauda epididymis were fixed in 2.5% glutaraldehyde overnight. Transmission electron microscopy was performed by the Electron Microscopy Laboratory of Nanjing Medical University. Briefly, the sperm were post-fixed with 2% OsO4 and embedded in acrylate resin. Samples were stained with uranyl acetate and lead citrate and observed under a JEM-1010 transmission electron microscope (JEOL, Tokyo, Japan).

### Terminal deoxynucleotidyl transferase dUTP Nick-End Labeling (TUNEL) assay

For the analysis of apoptosis during spermatogenesis, TUNEL assay was performed on testicular paraffin sections from 8-week-old male mice using the TUNEL BrightRed Apoptosis Detection Kit (Vazyme, A113-01). The procedures were carried out according to the manufacturer’s instructions. Fluorescence was detected using an LSM800 confocal laser scanning microscope (Zeiss, Oberkochen, Germany). A minimum of 100 seminiferous tubules were counted per testis (5 sections/testis) and three animals were examined.

### Immunofluorescence

Elongated spermatids from the testis and sperm from the cauda epididymis were spread onto microscope slides to air-dried and fixed in 4% paraformaldehyde for 1 h, then washed three times with PBS. The sperm samples were sequentially blocked with 5% bovine serum albumin (BSA) containing 0.1% triton X-100 for 2 h at room temperature, and incubated overnight at 4 °C with primary antibodies. Next day, the slides were incubated with secondary antibodies for 1 h at room temperature. After being counterstained with PNA (Merck, L7381) and Hoechst 33342 (Thermo, H1399), the slides were visualized with an LSM800 confocal microscope (Zeiss, Germany).

### Chromosome spreads

Seminiferous tubules were cut into pieces with scissors, and incubated in 0.4% NaCl solution. Cell suspensions were fixed with fixative solution (1% paraformaldehyde, 0.15% Triton X-100 and 0.5 M sodium borate). Samples were blocked with 1 × ADB (1% normal donkey serum, 0.03% BSA, and 0.05% Triton X-100) for 1 h at room temperature and incubated with primary antibodies at 37 °C for 12–16 h. The next day, the samples were blocked with 1x ADB for 2 h at room temperature and incubated with secondary antibodies at 37 °C for 1.5 h. The slides were viewed under an LSM800 confocal microscope (Zeiss, Germany).

### Protein extraction and Western blotting

Proteins from cultured cells and testicular tissues were extracted using RIPA lysis buffer (Beyotime, P0013C) containing 1% protease and phosphatase inhibitors (NCM Biotech, P002). The protein samples were mixed with loading buffer and boiled for 5 min. The denatured proteins were separated by 10% or 12% SDS-polyacrylamide gels and transferred onto nitrocellulose membranes (Bio-Rad, 1620177). The membranes were blocked with 5% BSA (Beyotime, ST205) for 2 h at room temperature and then incubated overnight with the primary antibody at 4 °C. The next day, the membranes were washed and incubated for 1 h with horseradish peroxidase-conjugated secondary antibodies. The signals were detected by a Bio-Rad gel imaging system. The gray values of the bands were analyzed by ImageJ software. The antibodies used are listed in Appendix Table S1.

### Cell culture and plasmid transfection

HEK293T cells were cultured in Dulbecco’s modified Eagle’s medium (DMEM) (Biochannel, BC-M-032) supplemented with 10% fetal bovine serum (FBS) (Biochannel, BC-SE-FBS01), 1% penicillin–streptomycin solution (Biochannel, BC-CE-007) at 37 °C in a humidified 5% CO_2_ incubator. The coding sequence of target protein was PCR-amplified from a mouse testis cDNA pool, and the PCR products were purified using the FastPure Gel DNA Extraction Mini Kit (Vazyme, DC301-01). The PCR products were cloned into the pcDNA3.1(+) plasmid with a Flag tag using ClonExpress II one-step cloning kit (Vazyme, C115-01). Point mutation plasmids were constructed using the Mut Express II Fast Mutagenesis Kit (Vazyme, C214-01). For plasmids transfection, HEK293T cells were transfected with the plasmid using ExFect Transfection Reagent (T101-01) and cultured for 36-48 h.

### Immunofluorescence staining of PI(4,5)P_2_

Cells were fixed with 3% glutaraldehyde for 3 h on ice. All subsequent steps were performed either on ice or at 4 °C. After fixation, the samples were rinsed thrice with 50 mM NH_4_Cl (Beyotime, ST2030-100g) in PBS and blocked for 4 h with 0.5% saponin (Beyotime, ST2618-5g), 5% normal goat serum (Sangon Biotech, E510009) and 50 mM NH4Cl in NaGB (20 mM PIPES-NaOH, 137 mM sodium glutamate, 2 mM MgCl2, 1 mg/mL BSA). This blocking solution was then removed and replaced with anti-PtdIns(4,5)P2 antibody 2C11 (Echelon Biosciences, Z-P045) overnight at 4 °C. Next day, the slides were incubated with secondary antibodies for 1 h at room temperature, and visualized using an LSM800 confocal microscope (Zeiss, Germany). To measure the level of PI(4,5)P_2_ in HEK293T cells, the fields of view were randomly selected and photographed under identical conditions. The signal intensity was analyzed using Image J.

### Malachite Green Assay

In vitro lipid and protein phosphatase assays were performed with a malachite green phosphatase assay kit (Echelon Biosciences, K-1500) to measure TPTE activity. Following the manufacturer’s instructions, C-TPTE or TPTE-Mut proteins were immunoprecipitated from the transfected cell lines with Anti-Flag Magnetic Beads (Beyotime, P2115) for overnight at 4 °C. The protein-bound beads were washed three times with 1×TBS and twice with reaction buffer (1 × TBS, 10 mM DTT). The beads were incubated with the respective 1 mM PI(3,4,5)P_3_ (Echelon Biosciences, P-3908) in a reaction buffer at a 25 μl scale for 1 h at 37°C. Malachite green solution was then added to terminate the enzyme reaction and incubated for 20 min at room temperature. The released phosphate was quantified by measuring absorption spectrum at 620 nm using a microplate reader (TD-20/20, Turner Designs, CA, USA) and the amount converted to free phosphate was calculated.

### Separation of mouse testicular cells

Testes were decapsulated and digested with collagenase IV digestion solution (Gibco, 17104-019) in DMEM (Gibco, 11995073) for 15 min at 37 °C. The seminiferous tubules were obtained by leaving the sample on ice for 5 min. Collect the supernatant, and then the interstitial cells will be obtained by centrifugation at 800 × *g* for 5 min. Digest the seminiferous tubules with 0.25% trypsin including 1 mg/mL DNase I (Gibco, 25200072) for 10 min. The digestion was terminated by adding DMEM with 10% FBS, and the single-cell suspensions were washed twice with DMEM solution.

Single-cell suspension was cultured in DMEM/F12 (Thermo, A4192001) supplemented with 10% FBS (Biochannel, BC-SE-FBS01), 1% penicillin–streptomycin solution (Biochannel, BC-CE-007) at 37 °C in a humidified 5% CO_2_ incubator. Change the culture medium every 24 h, and Sertoli cells adhering to the bottom of the dish were collected.

Spermatogonia were isolated from postnatal day 8 mouse testes through STA-PUT method [54]. Single-cell suspensions were further loaded into a cell separation apparatus (ProScience Inc. Toronto, Canada) and followed by 2–4% BSA gradient. After the cells have settled for 2 h, the target cell fraction was collected. Spermatocytes, round spermatids and elongated spermatids were isolated from adult mouse testes and collected by using the JE-5.0 elutriator systems (Beckman, CA, UAS) according to the manufacturer’s instruction [55]. In brief, the entire instrument was rinsed with EKRB buffer (120.1 mM NaCl, 4.8 mM KCl, 25.2 mM NaHCO_3_, 1.2 mM KH_2_PO_4_, 1.2 mM MgSO_4_, 1.3 mM CaCl_2_, 11 mM glucose), then the cell suspension was slowly injected into the pump to 13.5 mL/min and set the rotor at 2800 × *g*. After 350 mL of liquid is collected to obtain elongated spermatids, speed up the flow rate to 31.3 mL/min, and continue to collect 150 mL cell suspension to obtain round spermatids. Set the rotor at 2800 × *g* and the flow rate to 40 mL/min to collect the spermatocytes. Immunofluorescence analyzed at least 200 cells and detected purity above 80%.

### High-pH reverse-phase fractionation

For protein quantification, TMT-labeled peptide mixture was fractionated using ACQUITY UPLC M-Class with XBridge BEH C18 column (300 μm × 150 mm, 1.7 μm; 130 Å, Waters). A 128 min gradient (3% buffer B for 14 min, 3–8%B for 1 min, 8–29%B for 71 min, 29–41% B for 12 min, 41–100% B for 1 min, 100% buffer B for 8 min, 100–3% B for 1 min, followed by 20 min at 3% B) was employed with buffer A (20 mM ammonium formate, pH 10) and buffer B (100% ACN). 30 fractions were collected using nonadjacent pooling scheme and then dried with a SpeedVac concentrator (Labconco, MO, USA).

### Mass spectrometry analysis

Testicular cells were extracted by protein extraction buffer (8 M urea, 75 mM NaCl, 50 mM Tris, pH 8.2, 1% EDTA-free protease inhibitor, 1 mM NaF, 1 mM β-glycerophosphate, 1 mM sodium orthovanadate, 10 mM sodium pyrophosphate), followed by reduction, digestion, and desalting. Peptides were resuspended in 0.1% formic acid and analyzed with an LTQ Orbitrap Fusion Lumos mass spectrometer (Thermo Finnigan) coupled to the Easy-nLC 1200. A 95-min linear gradient (3% to 5% buffer B for 5 s, 5% to 15% buffer B for 40 min, 15% to 28% buffer B for 34.8 min, 28% to 38% buffer B for 12 min, 30% buffer to 100% buffer B for 5 s, and to 100% buffer B for 8 min) was applied at 300 nl/min. The Orbitrap Fusion Lumos was configured with a resolution of 60 K for MS1 and 15 K for MS/MS, and the data were acquired by Xcalibur 4.3.73.11 (Thermo Fisher Scientific).

All raw files were searched with MaxQuant software (Version 2.2.0.0) using mouse protein sequences obtained from the Universal Protein Resource database (UniProt). Both the protein and peptide FDR cut-off was set to 0.01. The corrected TMT reporter intensities were used for TMT-based protein quantification. Statistical significance was determined using the unpaired two-tailed Student’s *t* test. A protein with *P* value < 0.05 and fold change > 2 was considered significantly differentially expressed. Then, significantly upregulated proteins were run using the functional annotation tool of the Database for Annotation, Visualization, and Integrated Discovery (DAVID).

### Sucrose Density Gradient Centrifugation

Round spermatids were washed with ice cold PBS and lysed with 300 μl of polysome lysis buffer (100 mM KCl, 2 mM MgCl_2_, 10% glycerol, 50 mM HEPES, 0.1% Triton X-100, 1 mM DTT, 20 U/mL RNase inhibitor (Promega, N2511), 1×cocktail (Thermo, 87785) and 100 mg/mL cycloheximide). After lysis of the cells by gently blowing, cell debris was removed by centrifugation (10,000 × *g*, 15 min, 4 °C). The supernatant was then layered onto a 13.2 mL linear sucrose gradient 15%–55% (w/v), supplemented with 100 mM NaCl, 5 mM MgCl_2_, 10 mM Tris-HCl (pH 7.5) and 1 mM DTT and centrifuged (26 000 ×g, 2.5 h, 4 °C) using a Beckman SW41Ti rotor (Beckman). Approximately 30 fractions from each condition were collected using a gradient station (Biocomp, Canada) with continuous measurement of absorbance at 254 nm.

### Statistical analysis

Statistical analysis were evaluated using Graphpad Prism 10. The data are presented as the mean ± standard deviation (SD) of at least three biological and technical triplicates tests. For multi-group comparison, such as gene expression data, intensity of PI(4,5)P_2_ and fertility analysis, one-way ANOVA followed by Dunnet’s multiple comparison test was used. For comparison between two groups, including spermatogenic cell counts, Western blot quantification data, and sperm parameters by Computer Assisted Sperm Analysis, two-tailed Student’s *t* test was used. *P* < 0.05 was considered significant.

## Supplementary information

Supplementary information

Appendix Figure S1

Appendix Figure S2

Appendix Figure S3

Appendix Table S1

Appendix Table S2

Appendix Table S3

## Data Availability

All the raw data of proteomics are available in supplementary information.
