# Supplementary material for: TPTE, a testis-specific PTEN family member, drives spermatogenesis via PI(4,5)P2 synthesis
Source: Cell Death Dis. 2026 Mar 25;17(1):378. doi: 10.1038/s41419-026-08614-3 (PMC13039310; doi:10.1038/s41419-026-08614-3)
Supplement: Supplementary file 1 — Supplementary information [file 41419_2026_8614_MOESM1_ESM.docx]

**Supplementary information**

**Appendix Figure S1. Expression analysis of *Tpte* mRNA. (A)** Design of knockout verification primers. **(B)** RT-PCR verification of the deletion-induced *Tpte* cDNA frameshift mutation. **(C)** qRT-PCR detection of *Tpte* mRNA expression level in testes (n=3, two-tailed Student’s *t*-test). ***P*<0.01.

**Appendix Figure S2.** **Morphological analysis of *Tpte^-/-^* epididymis.** The morphology (A) and epididymis/body weight ratio (B) of *Tpte^+/+^* and *Tpte^-/-^* mice (n=5, two-tailed Student’s *t*-test). ns, not significant.

**Appendix Figure S3. Morphological analysis of** ***Tpte^-/-^* testes. (A-B)** The morphology (A) and testis/body weight ratio (B) of *Tpte^-/-^* and control testes (n=5, two-tailed Student’s t-test). **(C-D)** The periodic acid-Schiff (PAS) staining (C) and stages of seminiferous epithelial cycle of *Tpte^+/+^* and *Tpte^-/-^* testes. A, type A spermatogonia; In, intermediate spermatogonia; B, type B spermatogonia; Pl, Preleptotene; L, leptotene; Z, Zygotene; P, Pachytene; D, Diplotene; MI, metaphase I; Rst, Round spermatid; Est, Elongated spermatid. Bar, 50μm. ns, not significant.

**Appendix Table S1.** Antibodies used in this study.

**Appendix Table S2.** Differentially expressed proteins in *Tpte^-/-^* round spermatids.

**Appendix Table S3.** GO terms enriched in proteins up-regulated in *Tpte^-/-^* round spermatids.
