# Supplementary figures and images for: TPTE, a testis-specific PTEN family member, drives spermatogenesis via PI(4,5)P2 synthesis
Source: Cell Death Dis. 2026 Mar 25;17(1):378. doi: 10.1038/s41419-026-08614-3 (PMC13039310; doi:10.1038/s41419-026-08614-3)

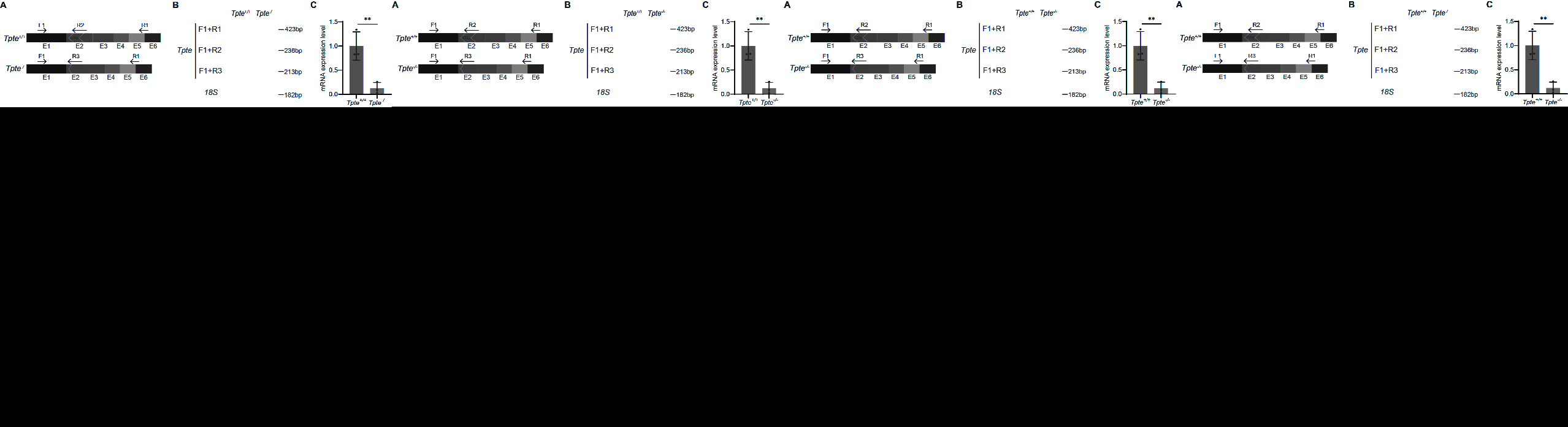

Supplement: Supplementary file 2 — Appendix Figure S1 [file 41419_2026_8614_MOESM2_ESM.tif]

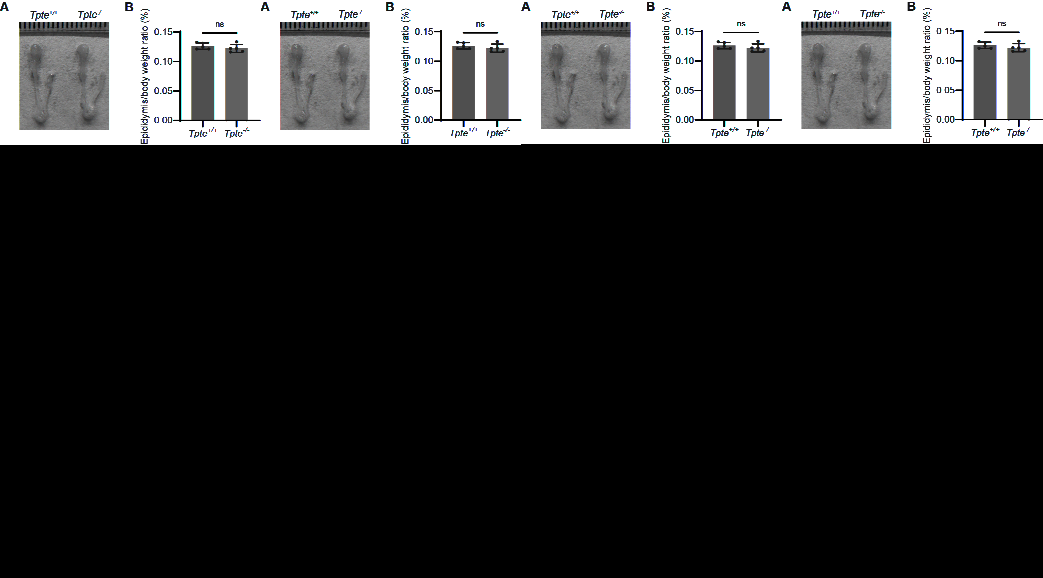

Supplement: Supplementary file 3 — Appendix Figure S2 [file 41419_2026_8614_MOESM3_ESM.tif]

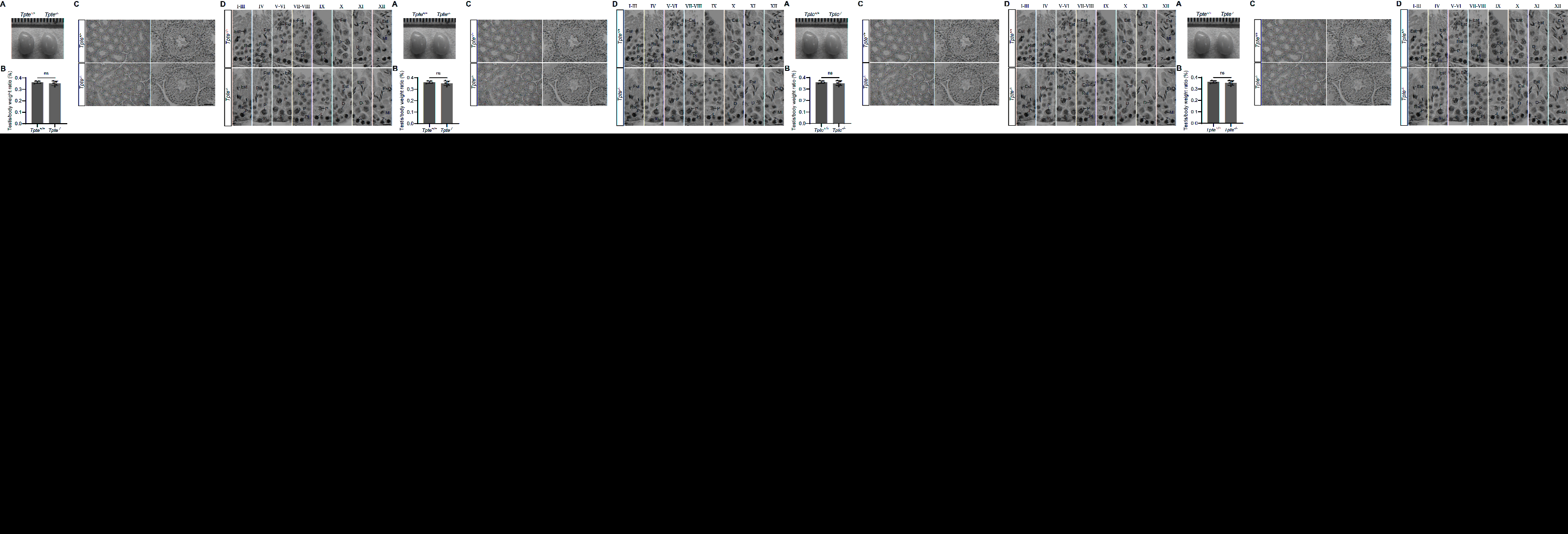

Supplement: Supplementary file 4 — Appendix Figure S3 [file 41419_2026_8614_MOESM4_ESM.tif]
